# Supplementary material for: Identification of Environmental Determinants Involved in the Distribution of Burkholderia pseudomallei in Southeast Asia using MaxEnt software
Source: PLoS Negl Trop Dis. 2025 Jan 7;19(1):e0012684. doi: 10.1371/journal.pntd.0012684 (PMC11741614; doi:10.1371/journal.pntd.0012684)
Supplement: S1 Table — (DOCX) [file pntd.0012684.s001.docx]

**Supplementary Table 1: SEA Countries with High to Very High suitability to Bp*.***

| **Country** | ***Predicted Areas to be Suitable to Burkholderia pseudomallei and their Location Names*** | | |
| --- | --- | --- | --- |
| **Cambodia** | Oddar Meanchey  Kampong Thom  Kampong  Chhnang  Koh Kong  Sihanoukville  Stung Treng  Ratanakiri  Mondulkir | **Myanmar** | Bawlakhe District, Kayah  Sittwe District, Rakhine  Mrauk-U District, Rakhine  Kyaukse District, Mandalay  Maungdaw District, Rakhine  Kanbalu District, Sagaing  Katha District, Sagaing  Hkamti District, Sagaing  Mawlaik District, Sagaing |
| **Indonesia** | South Papua  Maluku  Southeast Sulawesi  Gorontalo  East Nusa Tenggara  East Kalimantan | **Thailand** | Songkhla Province  Krabi Province  Surat Thani Province  Prachuap Khiri Khan Province  Samut Sakhon Province  Chai Nat Province  Kamphaeng Phet Province  Sa Kaeo Province  Maha Sarakham Province  Loei Province  Buriram Province  Kalasin Province  Udon Thani Province  Nakhon Phanom Province  Si Sa Ket Province  Yasothon Province  Bueng Kan Province  Ubon Ratchathani Province  Kanchanaburi Province  Uttaradit Province |
| **Laos** | Bolikhamsai  Salavan  Khammouane  Attapu |  |  |
| **Malaysia** | Petaling Jaya, Selangor  Ipoh, Perak  Kedah |  |  |
| **East Timor** | Metinaro |  |  |
| **Vietnam** | An Giang Province  Thu Duc City  Binh Thuan Province  Ninh Thuan Province  Khánh Hòa Province  Phu Yen Province  Tuyen Quang Province  Bình Định Province  Nghệ An Province  Thanh Hoa province  Ninh Binh Province  Thai Nguyen Province  Bac Giang Province  Quang Ninh Province | **Philippines** | Oriental Mindoro, Mimaropa  Palawan, Mimaropa  Negros Occidental, Western Visayas  Tarlac, Central Luzon  Nueva Ecija, Central Luzon  Zambales, Central Luzon  Abra, CAR  Maguindanao, ARMM  Negros Oriental, Central Visayas  Pangasinan, Ilocos Region  Sarangani, Soccsksargen  Ilocos Sur, Ilocos Region  Ilocos Norte, Ilocos Region  Pampanga, Central Luzon  General Santos, Soccsksargen |
