# Supplementary material for: Identification of Environmental Determinants Involved in the Distribution of Burkholderia pseudomallei in Southeast Asia using MaxEnt software
Source: PLoS Negl Trop Dis. 2025 Jan 7;19(1):e0012684. doi: 10.1371/journal.pntd.0012684 (PMC11741614; doi:10.1371/journal.pntd.0012684)
Supplement: Attachment — Submitted filename: Cover letter_Revised Manuscript_plos.pdf [file pntd.0012684.s002.pdf]

1 July 2024

DAWIT GEBEYEHU GETACHEW, MPH
Guest Editor
PLOS Neglected Tropical Diseases

STUART BLACKSELL
Section Editor
PLOS Neglected Tropical Diseases

Dear Editors,

On behalf of the authors, I am resubmitting our manuscript with the revisions based on the reviewers' comments and questions. These are addressed point-by-point and as follows.

I. **Key Review Criteria Required for Acceptance?**
As you describe the new analyses required for acceptance, please consider the following:

A. **Methods**
-Are the objectives of the study clearly articulated with a clear testable hypothesis stated?
-Is the study design appropriate to address the stated objectives?
-Is the population clearly described and appropriate for the hypothesis being tested?
-Is the sample size sufficient to ensure adequate power to address the hypothesis being tested?
-Were correct statistical analysis used to support conclusions?
-Are there concerns about ethical or regulatory requirements being met?

Reviewer #1: Yes

Reviewer #2: Yes, the hypothesis is understandable. Methods could be greatly improved. As a reader, I was quite confused as important terms are not sufficiently defined in the methods section. AT the same time, large parts of the methods text could be summarized more succinctly. The authors copy/paste command/settings: Lines 198-201 for previously published tools.

Authors' Response: We sufficiently defined the important terms and succinctly summarized the method section. We also addressed the confusion in lines 173, 186 - 188, 198 - 201.

I also suspect that an entire figure was accidentally excluded or mis-referenced. In Line 173, the authors explain how a subset of the bioclimatic variables were excluded, but the figure referenced is a map. The authors miss an opportunity to clearly communicate how the tools were used. Each was previously published and open source, but sentences like Line 186 to 188 are not clearly stated, and technically confusing.

Authors' Response: We corrected the Figure that was misplaced and instead placed a Table 1 showing all the factors being mentioned in the text. Lines 186-188 was also shortened to make clearer statements.

Reviewer #3: (No Response)

B. **Results**

-Does the analysis presented match the analysis plan
-Are the results clearly and completely presented?
-Are the figures (Tables, Images) of sufficient quality for clarity?

Reviewer #1: Yes

Reviewer #2: From what I can tell, yes, the analysis matches, but the authors again miss a big opportunity to clearly communicate their findings, instead of relying on overly technical vocabulary and jargon. The figure legends are frustratingly brief and at times confusing (Fig 3). Alone, the figure legend is unable to summarize the what the figure communicates (is this the final species distribution model being tested?). The results section is an opportunity for the authors to describe the env factors that did not appear to contribute to their assessment of habitat suitability? The other factors are ignored and seemingly deemed unimportant. The authors tell an incomplete story, and leave the reader frustrated by the lack of transparency.
The authors must revisit each figure legend and consider writing sentences that help the reader understand what is captured. Figure 4 mentions "response curve for Bp to annual precipitation" which could lead to misunderstanding what is being tested, because the authors are predicting the usefulness of data here. It could be easily confused that the authors have expose B.pseudomallei to different amounts of rain.

Authors' Response: We revisited all the figures and provided the necessary descriptions on the figure legends to convey the message for each figure. Furthermore, we clarified what is being communicated in Figure 4 and made the necessary corrections.

Reviewer #3: (No Response)

C. **Conclusions**

-Are the conclusions supported by the data presented?
-Are the limitations of analysis clearly described?
-Do the authors discuss how these data can be helpful to advance our understanding of the topic under study?
-Is public health relevance addressed?

Reviewer #1: Yes

Reviewer #2: The authors miss an opportunity to clearly state their study findings. The first sentence repeats jargon and nomenclature that assumes the reader runs these analyses routinely (Bio8, Biocateg1, Bio12), instead of communicating how these are translated. As a scientist who had never run these analyses, the authors have missed an opportunity to translate the implications of their findings to the broad public health and scientific community. Lines 337 to 340 as standalone sentences if re-written, could belong in the results section, but are overly technical. How do the authors translate this permutation importance for public health? Again, in lines 360-362, the authors hold fast to code-words (Biocateg1, Bio8, Bio12) with zero context for the reader. It's not until Line 375-78 that the authors reveal the code--this sentence should begin the discussion section, but instead this is nearly the final sentence of the report.

Reviewer #3: (No Response)

Authors' Response: We spelled out all the significant environmental variables mentioned in our manuscript with the corresponding abbreviations, instead of using the acronyms coming from the software. The entire discussion section has been revised for clarity and coherence.

D. **Summary and General Comments**

Use this section to provide overall comments, discuss strengths/weaknesses of the study, novelty, significance, general execution and scholarship. You may also include additional comments for the author, including concerns about dual publication, research ethics, or publication ethics. If requesting major revision, please articulate the new experiments that are needed.

Reviewer #1: Overall, this is a very well written and interesting manuscript.

Major: nil
Minor:
The abstract is well written. Does it need to have subheadings?
Line 128: prolonged
Line 173: should this be Supplementary Table 1 and not "Figure 2"
The use of Biocateg1, Bio8, or Bio12, can be difficult to follow while reading the manuscript. Could the authors please spell out what these factors are in the text instead. E.g. line 230: "Bio8 contributed 67·9% probability of suitability, followed by Biocateg1 and then Bio12"
Line 315: can the authors expand on why they think regions with precipitation >2300mm/year would have less B. pseudomallei? Would this same explanation hold true for flood events?

Reviewer #2: All comments are captured above. The authors miss a huge opportunity to

communicate their findings and the implications of their study results. Unfortunately, this study report is overly technical and relies on jargon and would benefit from sharing the discussion text with colleagues who do not perform these software models routinely. I strongly urge the authors to revisit each figure and legend, the results section and the first paragraph of the discussion section. Consider whether the text could be clearer for the readership of this journal.

Reviewer #3: Dear Editor,
The manuscript titled "Identification of Environmental Determinants Involved in the Distribution of Burkholderia pseudomallei in Southeast Asia using MaxEnt software" investigates the environmental variables predicting the suitable ecological niche for Burkholderia pseudomallei (Bp), a bacterium causing melioidosis, in Southeast Asia. Using MaxEnt modeling combined with systematic reviews and meta-analysis, the study identifies bioclimatic and edaphic factors as key determinants of Bp's distribution. The most influential variables include the mean temperature of the wettest quarter (≥26°C), annual precipitation (<2300 mm), and Acrisol soil type. The study produces a distribution map highlighting areas with high suitability for Bp presence, significantly contributing to public health planning and disease burden assessment in the region under current climatic conditions.

It is a well-written manuscript. While the manuscript seems thorough and scientifically robust, there are a few limitations that could be considered:

1. Geographic Scope: The study focuses specifically on Southeast Asia. While this is a strength in terms of depth, it may limit the applicability of the findings to other regions where Burkholderia pseudomallei is found, such as Northern Australia and other tropical areas. Extending the study to include these regions could provide a more comprehensive understanding of the environmental determinants.

   Authors' Responses:

   a. **The scope of the regional area under study was focused only on Southeast Asia (SEA) where there is a relative commonality in weather patterns, hydroclimatic processes, geography and anthropogenic activities. Even the recent intense heatwaves were solely experienced by SEA and the seasonal monsoon thereafter.**
   b. **The region is roughly at the same level of economic development that has experienced a significant shift not only in land use but also temporal demographic changes. That excludes the rampant illegal animal pouching and trade in this region.**

2. Model Dependence: The study heavily relies on MaxEnt software for ecological niche modeling. While MaxEnt is a widely used and robust tool, the results are inherently

dependent on the assumptions and limitations of the model, such as the reliance on presence-only data and the potential for overfitting, especially with a large number of predictors.

Authors' Response: This is correct. We have incorporated in our revised text such limitations of using the MaxEnt algorithm.

3. Data Availability: The manuscript mentions that all research data can be accessed upon request, which could limit immediate verification and replication of the study results by other researchers. Making data openly available in a recognized repository could enhance transparency and reproducibility.

   Authors' Response: We already deposited our generated metadata in the journal-recognized public repository and provided the accession numbers in the data availability statement mentioned in the manuscript.

4. Environmental Variables: The selection of environmental variables, while extensive, might still omit other potentially influential factors that could affect the distribution of *Burkholderia pseudomalle*i. For instance, human activity or land-use changes, which are significant in tropical regions, could also impact the bacterium's distribution but are not included in the model.

   Authors' Response: For this study, we have only focused on the correlative nature of the MaxEnt modeling. Hence, we only utilized factors that we have easily accessed with such as the bioclimatic factors and topographic/edaphic factors. For a more mechanistic approach, another phase of the modeling study the authors intend to do. We have plans of exploring the potentialities of incorporating such anthropogenic data if available.

5. Static Time Frame: The environmental data used for the model are presumably static or represent average conditions. This does not account for year-to-year climatic variability or long-term climate change, which could significantly affect the distribution patterns of the pathogen.

   Authors' Response: This is another limitation of such model implementation that the authors highlighted in the section of the discussion of the revised manuscript, since the data available for the models comes from databases that are of static in time frame. Nevertheless, studies that mostly employed the same algorithm used similar data for the predictive ability of the models.

6. Confirmation of Model Predictions: While the model predicts potential distributions based on environmental suitability, actual occurrences of Burkholderia pseudomallei would need to be confirmed through field sampling and laboratory confirmation, which could provide a robust test of the model's accuracy.

Addressing these limitations in future research could provide a more nuanced understanding of the factors influencing the distribution of Burkholderia pseudomallei and potentially improve the predictive capabilities of the models used.

Authors' Response: We certainly agree that ground truthing of the result will have a more convincing power and reliability for the model generated by the study. Such limitation was again highlighted in the revised version and such ground truthing is intended to be conducted in a more directed and strategized sampling the authors would be doing as part of the next phase of the research study.

We hope that the reviewers' comments and questions have been sufficiently addressed point-by-point and the corresponding revisions made in the manuscript for improvement and clarity.

Thank you very much for your time and consideration.

Sincerely yours,

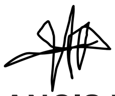

JOSE FRANCIS V. ABRANTES
Corresponding Author
