# Supplementary material for: Identification of Environmental Determinants Involved in the Distribution of Burkholderia pseudomallei in Southeast Asia using MaxEnt software
Source: PLoS Negl Trop Dis. 2025 Jan 7;19(1):e0012684. doi: 10.1371/journal.pntd.0012684 (PMC11741614; doi:10.1371/journal.pntd.0012684)
Supplement: Attachment — Submitted filename: Cover letterII_Revised Manuscript_plos.docx [file pntd.0012684.s003.docx]

11 October 2024

**DAWIT GEBEYEHU GETACHEW**, MPH

Guest Editor

PLOS Neglected Tropical Diseases

**STUART BLACKSELL**

Section Editor

PLOS Neglected Tropical Diseases

Dear Editors,

On behalf of all authors, I am resubmitting our manuscript with the revisions based on the reviewers’ comments and suggestions. These are addressed as follows.

A. **Summary and General Comments**
   Use this section to provide overall comments, discuss strengths/weaknesses of the study, novelty, significance, general execution and scholarship. You may also include additional comments for the author, including concerns about dual publication, research ethics, or publication ethics. If requesting major revision, please articulate the new experiments that are needed.

   Reviewer #1: I believe the authors have responded appropriately to the majority of the reviewer comments.
   I have included a few minor comments to help improve the readability of the manuscript.
   - done in the revised version

Abstract:
The use of (Bio8) and (Bio12) in the abstract is somewhat unhelpful without prior explanation.

- done in the revised version

Line 260-262: it would be easier for the reader to interpret these sentences if the “Bio” included the associated factor just in this section when they are 1st mentioned in the main text e.g. Bio8 (mean temperature…) ; Biocat1 (soil type).

- done in the revised version

Line 276: cloglog, technically an abbreviation and should be spelled out on first use.

- done in the revised version

Line 340: Melioidosis doesn’t need to be capitalized

- done in the revised version

Line 386: check grammar/syntax

- done in the revised version

Line 438: remove “even”

- done in the revised version

**Authors’ Responses: We corrected and clarified the items mentioned above.**

Reviewer #3:

Dear Editor,

The revised manuscript has been significantly improved and addressed most of the reviewers' comments and questions. However, I have a request that the authors should consider including their response to my concern #4 regarding the environmental variables below to the revised manuscript. This is a limitation of using the current methods and this limitation needs to be documented.

Environmental Variables: The selection of environmental variables, while extensive,
might still omit other potentially influential factors that could affect the distribution of
*Burkholderia pseudomallei*. For instance, human activity or land-use changes, which
are significant in tropical regions, could also impact the bacterium’s distribution but are
not included in the model.

**Authors’ Response:** **We have restated and added another line to emphasize the correlative nature of our model and take into consideration that further investigations using a more mechanistic approach integrating land use and land cover. Anthropogenic stressors will also be added.**

PLOS authors have the option to publish the peer review history of their article. What does this mean? If published, this will include your full peer review and any attached files.

If you choose “no”, your identity will remain anonymous, but your review may still be made public.
Do you want your identity to be public for this peer review?

Reviewer #1: No

Reviewer #3: Yes: Apichai Tuanyok

We hope that the reviewers’ comments and suggestions have been sufficiently addressed in the manuscript for improvement and clarity.

Thank you very much for your time and consideration.

Sincerely yours,

sgd

**JOSE FRANCIS V. ABRANTES**

Corresponding Author
